# World Housing Conference

**Published:** 1920-06-12

**Authors:** 


### World Housing Conference.

### England as a Pioneer.

A discussion on housing in which delegates from England, America, France, Italy, Belgium, Spain, Sweden, Norway, Holland, Denmark, Switzerland, Finland, Greece, Chile, Uruguay, Yugo-Slavia, Czecho-Slovakia, Canada, New Zealand, Liberia, and India all spoke during one morning session seemed hardly a promising start—from the point of view of serious debate—for the week's Inter-Allied Housing Congress, which has just concluded at Westminster. But valuable business was done, and it was flattering to hear that British courage—"even recklessness"—in handling the problem is admired in other countries. The chief Swedish delegate said they were trying to follow Britain in the big fight against the greatest social evil of the times, and the French Minister of Health took a similar attitude. Dr. Addison, who presided at the opening session, laid emphasis on the vital social and political importance of vigorous action; the Prime Minister sent a letter on the same lines, and the general feeling of the Congress certainly showed a clear comprehension of that fact. Every nation, especially those who have been at war, has suffered either through devastation, dislocation of industry, shortage of labour and material, and the virtual cessation of building during the war period. Badly as England is situated, many other lands are in much worse case, and have not even prepared national schemes. We have at least a scheme, detailed house plans for 187,000 houses, tenders sanctioned for 107,000, approval for 48,000 acres of land as sites for houses, and for nearly 4,000 lay-out schemes. Many delegates, who are to see for themselves how these plans are developing and what sort of houses are being erected, obviously came to England for information and a lead, and this is doubtless surprising news for the critics of the Government. It must in fairness be placed to the credit of the Government, however slow we may consider they have been. Dr. Addison hopes to have a large number of houses built before the end of the present year.

State and Private Enterprise.

A resolution was passed (against a small minority, which included an American delegate and a representative of the English Miners' Federation) urging legislative action by each Government in the preparation of a national housing policy of sufficient scope to secure within the limit of twenty years the proper housing of every family. The small opposition arose on the question of enlisting private enterprise, and in reply to this a good point was made by a Swedish delegate that the Socialist Swedish Government proposed to carry out its policy in co-operation with municipal authorities and private enterprise. The Commissioner for Health in New York City, speaking in support of the resolution, said he had recently made a survey of over 30,000 tenement houses in New York, and he found that tenements originally intended for five families were now housing ten families. There were hundreds of tenements in New York where twelve persons were living in three rooms, and where four persons slept in the kitchen every night. If the Government did not intervene in such circumstances there would be Bolshevism and crime, and in America they were desirous of getting the municipal authorities to provide funds to erect buildings. It is, of course, absurd to pretend that conditions like these are not the concern of the State, when it is remembered that the well-being of the State is based upon family life, and it is equally foolish to ignore the importance of private enterprise. The international Congress should prove valuable in the interchange of information and ideas which it made possible. For, judging from the first morning's experience, they showed no reluctance to speak.

